# Orthopedic infections associated with distinct *Acinetobacter* strains in rural area of Qingdao, China

**DOI:** 10.3389/fcimb.2025.1601779

**Published:** 2025-07-30

**Authors:** Ying Wang, Dan Zhao, Yeshun Fan, Yanxiang Cui, Yingdi Wang, Xiaoxuan Guan, Linhong Yu, Shunqian Yuan, Lan Wang, Jianqiang Hu, Yisong Li, Wenbo Xia, Jie Liu

**Affiliations:** ^1^ School of Public Health, Qingdao University, Qingdao, Shandong, China; ^2^ Department of Clinical Laboratory, Qingdao Huangdao District Traditional Chinese Medicine Hospital, Qingdao, Shandong, China; ^3^ Qingdao Medical College, Qingdao University, Qingdao, Shandong, China; ^4^ Department of Orthopedics, Qingdao Huangdao District Traditional Chinese Medicine Hospital, Qingdao, Shandong, China

**Keywords:** orthopedic infection, *Acinetobacter baumannii-calcoaceticus* complex, non-*A. baumannii* species, phylogenetic analysis, antibiotic resistance

## Abstract

**Introduction:**

*Acinetobacter baumannii* poses a profound global health threat because of multidrug resistance and its association with nosocomial infections. However, standard clinical diagnostics often report it together with other *Acinetobacter* species as *A. baumannii-calcoaceticus* complex (ABC), which unavoidably conceals the attribution of non-*A. baumannii* species. This study reported orthopedic infection cases associated with different *Acinetobacter* species and characterized the genomes of the culture isolates to evaluate their potential impact on the clinical treatment.

**Methods:**

Nine in-patients with *A. baumannii-calcoaceticus* complex identified by culture during hospitalization were enrolled by the Orthopedics Department from a local hospital in Qingdao, China. Their clinical data were reviewed. One ABC isolate from each patient was tested for drug susceptibility and subjected for whole-genome sequencing, followed by bioinformatic analyses.

**Results:**

Through whole-genome analysis, nine ABC isolates were identified as six *A. baumannii*, two *A. pittii*, and one *A. soli* with distinct antibiotic resistance profiles and phylogenetic characteristics, indicating progressing pathogen transmission across broad geographic regions in One Health perspective. All *A. baumannii* and *A. pittii* strains carried multidrug resistance genes, while *A. soli* bore only *amvA* and *rsmA*. Phenotypically, eight isolates were susceptible to almost all the antibiotics tested, with only one *A. baumannii* being multidrug resistant. Despite this, eight patients received cephalosporins following positive reports of *A. baumannii-calcoaceticus* complex.

**Conclusion:**

Our study highlighted the limitation of current clinical diagnostic approaches for non-*A. baumannii* cases, which tended to be overtreated, and suggested that *Acinetobacter* etiology landscape should be explored further beyond *A. baumannii* to avoid antibiotic misuse.

## 1 Introduction

Orthopedic infections associated with traumatic injuries have become increasingly prevalent in recent years (Chen et al., 2017; Metsemakers et al., 2024). It is challenging to promptly and accurately diagnose these infections due to their complex and nonspecific clinical manifestations, even when supported by well-established multidisciplinary approaches, including clinical assessments, such as leukocyte counts and inflammatory marker testing, microbiological methods, and histopathology analysis (Kavanagh et al., 2018; Glaudemans et al., 2019). Delayed or inaccurate diagnosis frequently impedes timely and appropriate treatment in many cases, leading to chronic infection, which is even more difficult to be cured and may result in disability or even death (Tande et al., 2022).

A substantial proportion of orthopedic infections are either hospital-acquired or surgery-related, and are particularly associated with a high risk of multidrug-resistant (MDR) pathogens, including *Acinetobacter baumannii* (Harding et al., 2018). *A. baumannii* is one of the most common healthcare-associated pathogens causing nosocomial pneumonia, bloodstream infections, and surgical-site or wound infections. It is notorious of widespread multidrug resistance or even pan-drug resistance, particularly threatening to immunocompromised or debilitated individuals, such as patients in intensive care units (ICU) (Lenie et al., 2007). *A. baumannii* together with *A*. *calcoaceticus*, *A*. *pittii*, and *A*. *nosocomialis* are generally acknowledged as *A. baumannii-calcoaceticus* complex (ABC), with limited routine efforts to further differentiate them at the species level in clinical practice (Nemec et al., 2011). Recent reports have highlighted the pathogenic potential of non-ABC *Acinetobacter* species, such as *A. soli*, but they have been largely overlooked due to inaccurate identification by conventional methods (Endo et al., 2014; Almuzara et al., 2015). The global dissemination of carbapenem-resistant *Acinetobacter* species is more concerning, which poses a significant threat to effective antimicrobial therapy and patient outcomes (Muller et al., 2023).

In our previous study, multiplex real time polymerase chain reaction (PCR) panels were employed to investigate the bacterial etiologies of orthopedic infections (Wang et al., 2024a; Wang et al., 2024b). *A. baumannii* was detected in approximately 2% of the infection cases using *A. baumannii*-specific qPCR. Interestingly, conventional culture methods identified a higher number of ABC cases, raising the possibility that many of the culture-positive but PCR-negative cases might be attributed to non-*A. baumannii Acinetobacter* species. Based on these findings, we hypothesize that orthopedic infections, previously attributed to the ABC, may to some extent involve under-recognized and clinically relevant non-*A. baumannii* strains, which tend to be over treated as drug resistant *A. baumannii*. Hence, this study characterizes *Acinetobacter* isolates obtained from orthopedic infection cases using whole-genome sequencing (WGS) to determine their species, resistance profiles, and virulence. By integrating genomic data, phenotype results, and clinical information, this study aims to improve the understanding of *Acinetobacter* diversity and genomic features in orthopedic infections, and thereby to support more accurate diagnosis and antimicrobial therapy.

## 2 Method

### 2.1 Study settings and ethics statement

This study included nine in-patients enrolled by the Department of Orthopedics at Qingdao Huangdao District Traditional Chinese Medicine Hospital in a rural area of Qingdao, China, between January 2021 and August 2024, meeting the case criteria as previously described (Wang et al., 2024a, 2024b). All patients were local residents with no recent travel history outside the region. Exudate specimens were collected upon clinical examination.

This study was conducted in accordance with the ethical principles of the 1964 Declaration of Helsinki and later amendments. Ethical approval was granted by the Ethics Committees of both Qingdao University and Qingdao Huangdao District Traditional Chinese Medicine Hospital on January 1, 2021. According to administrative policy at the time, no registration number was assigned.

### 2.2 Bacterial culture and antimicrobial susceptibility tests

Bacterial isolates were retrieved by streaking exudate swabs on blood agar plates (Babio, Jinan, China) and incubated at 37°C. Bacterial species identification and antimicrobial susceptibility tests were performed using the VITEK 2 COMPACT system (bioMérieux, France) according to the manufacturer’s constructions. The results were interpreted following the Clinical and Laboratory Standards Institute (CLSI) guidelines (M100, Ed31-35, Malvern, Pennsylvania, USA). The antibiotics tested included imipenem, meropenem, ampicillin/sulbactam, piperacillin/tazobactam, ceftazidime, cefotaxime, ceftriaxone, cefoperazone/sulbactam, cefepime, gentamicin, tobramycin, amikacin, ciprofloxacin, levofloxacin, trimethoprim/sulfamethoxazole, minocycline, polymyxin B, and tigecycline. *A. baumannii* ATCC 19606 was used as the quality control.

### 2.3 Whole-genome sequencing and bioinformatic analysis of clinical isolates

Whole-genome sequencing (WGS) was performed on one ABC isolate from each patient using the Illumina NovaSeq 6000 platform (Sangon Biotech, Shanghai, China), generating paired-end reads of 150 bp. Raw reads were quality-filtered and trimmed using Trimmomatic v0.36 to remove adapters and low-quality bases. Filtered reads were *de novo* assembled using SPAdes v3.15. Gene predictions and annotations were conducted using Prokka v1.14 with default bacterial settings. Phylogenetic analysis was performed for the nine clinical isolates along with publicly available genomes of *A. baumannii, A. pittii*, and *A. soli* downloaded from the National Center for Biotechnology Information (NCBI). These genomes were filtered using pyANI v0.2.10 to retain those with ≥ 95% genome-wide average nucleotide identity (ANI) to the clinical isolates under study. All protein-coding genes were clustered into orthologous groups (OGs) based on sequence similarity using MMseqs2 v13 with default parameters. Multiple sequence alignments were first generated at the protein level using MAFFT v7.475, and codon-based alignments were then obtained by back-translation using PAL2NAL v14. Poorly aligned or ambiguously aligned regions were removed with Gblocks v0.91. The resulting alignments were concatenated into a single dataset using in-house Python scripts. A maximum-likelihood phylogenetic tree was constructed using FastTree v.2.1 and visualized with the Interactive Tree of Life (iTOL).

Multilocus sequence typing (MLST) was performed via the pubMLST.org online platform using the *A. baumannii* scheme. Antibiotic resistance genes (ARGs) were identified using the Comprehensive Antibiotic Resistance Database (CARD) with the criteria “perfect and strict hits only”. Virulence factors of Pathogenic Bacteria database (VFDB) was used to screen for the presence of virulence genes (sequence identity ≥ 70%, query coverage ≥ 70%, E-value < 1e-5). Mobile genetic elements (MGEs) were identified with the following tools under default parameters: COPLA for plasmid classification, CRISPRCasFinder for CRISPR-Cas system detection, ISfinder for insertion sequences, MobileElementFinder for transposable elements (identity ≥ 90%, coverage ≥ 95%,E-value < 1e-5), and PHASTER for prophage prediction.

## 3 Results

### 3.1 Case description and phylogenetic characteristics of clinical isolates

**Figure 1:**
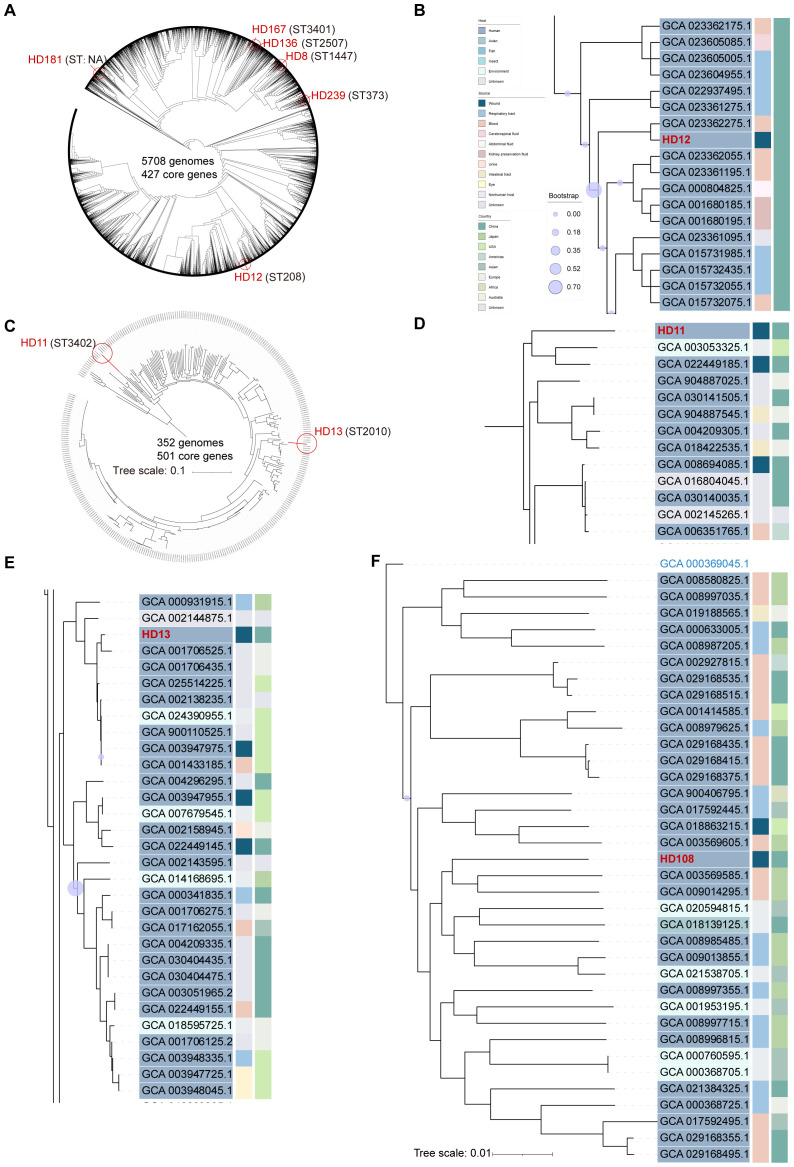
Phylogenetic analysis of *Acinetobacter* genomes identified in the current study. The phylogenetic trees were built using the maximum likelihood method based on 427 core genes and constructed by iTOL (https://itol.embl.de/) for 5701 whole genome sequences of *A*. *baumannii* with six *A*. *baumannii* isolates from the current study indicated by red circles, using *A*. *soli* CIP110264 as outgroup **(A)**. *A*. *pittii* HD11 and HD13, both highlighted by red circles, were plotted with 349 publicly available whole-genome sequences based on 501 genes, using *A*. *soli* CIP110264 as outgroup **(C)**. Subclades of HD12 **(B)**, HD11 **(D)**, and HD13 **(E)** were visualized respectively. *A*. *soli* HD108 was plotted with 35 reported genomes based on 800 genes with *A. pittii* ATCC19004 (GCA_000369045.1) as outgroup **(F)**. ST type of each strain is stated in parentheses. Tree scale bar represents 10% **(C)** or 1% **(F)** nucleotide sequence divergence, while the rest ignored branch lengths. All publicly available genomes were downloaded from NCBI and indicated with assembly accession numbers.

The nine cases included in this study had no overlapping hospital stays or evident epidemiological connection. Only Patient 1 shared a brief overlap with patients 2 and 7 during hospitalization, but their *Acinetobacter* isolates were phylogenetically distant or belonged to different species (**Table 1**, **Figure 1**).

**Table 1:** Demographic and clinical information of the enrolled patients in the current study.

Patient	Sex	Age (yr)	Admission date	Discharge date	Diagnosis upon admission*^a^*	Brief case description	Pathogen	Isolate number	ST	Sampling date	Clinical outcome
**P1**	Female	44	2021-02-17	2021-05-18	Traffic injury	8-day stay in the ICU, 90-day hospital stay, 8 times culture positive of ABC	*A. baumannii*	HD12 CRAB*^b^*	208	2021-03-14	De-sutured incisions, dry wounds, no exudate
**P2**	Male	71	2021-01-31	2021-02-20	Osteomyelitis	One-month history of purulent discharge, negative cultures upon admission	*A. baumannii*	HD8	1447	2021-02-09	Slight discharge
**P3**	Male	54	2022-10-05	2022-11-09	Traumatic injury	A fractured left femur after falling, skeletal traction in the general ward	*A. baumannii*	HD136	2507	2022-10-15	Infection not fully resolved*^e^*
**P4**	Female	73	2023-10-16	2023-11-20	Implant removal	Positive cultures from postoperative day 6	*A. baumannii*	HD167	3401*^c^*	2023-10-25	No signs of infection
**P5**	Male	54	2023-08-04	2023-08-21	Traumatic injury	An open-fractured finger with severe contamination, purulent exudates from postoperative day 8	*A. baumannii*	HD181	NA*^d^*	2023-08-16	Well-healed incision, no exudate
**P6**	Female	53	2024-06-17	2024-08-21	Traumatic injury	Hip replacement 40 days post injury, positive cultures from postoperative day 6	*A. baumannii*	HD239	373	2024-06-27	Well-healed incision, no exudate
**P7**	Male	67	2021-02-23	2021-03-05	Postoperative infection	One year post surgery, a 1-cm open wound in the right ankle with viscous drainage	*A. pittii*	HD11	3402*^c^*	2021-02-23	Slight discharge
**P8**	Female	81	2021-09-07	2021-09-13	Traumatic injury	Four days after trauma, skin and soft tissue infections	*A. pittii*	HD13	2010	2021-09-07	No exudate
**P9**	Male	59	2022-07-07	2022-08-11	Traumatic injury	Injured left extremity with contaminated deep wound	*A. soli*	HD108	NA*^d^*	2022-07-28	Skin and tissue defect with exudate*^e^*

*^a^* Clinical diagnosis upon admission.

*^b^* Carbapenem-resistant *A. baumannii* strain.

*^c^* Newly assigned sequence types.

*^d^* NA, not available.

*^e^* Despite the clinical symptoms, the patients insisted on being discharged against medical advice.

**Figure 2:**
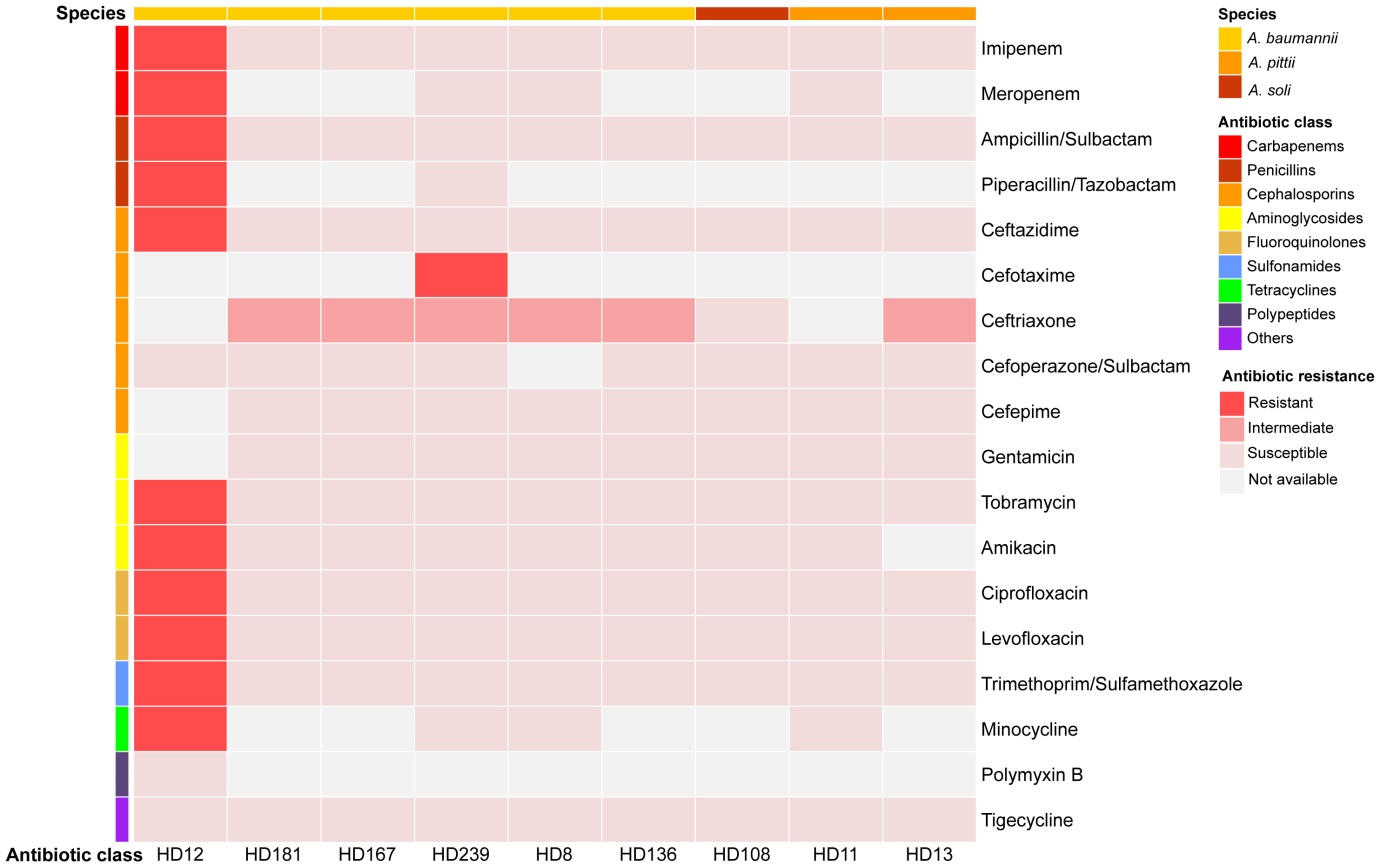
Antibiotic susceptibility profiles of the nine isolates. Results were visualized using Hiplot (https://hiplot.com.cn/).

Strains isolated from patients 1 to 6 were all initially identified as ABC by culture, and further clarified as *A. baumannii* by WGS (**Figure 1A**). Patient 1 was admitted to the ICU in critical condition following a severe traffic accident and underwent invasive skeletal traction upon arrival. She remained in the ICU for the first eight days and underwent five additional surgeries during a 90-day hospitalization. Yellowish, fish-smelling discharge was first observed at the fixation pinhole on day 9. Eight subsequent bacterial cultures from day 19 to 44 were all positive for ABC. The isolate included in this study, HD12, was obtained on day 26 and identified as carbapenem-resistant *A. baumannii* (CRAB) with a high level of multidrug resistance (**Figure 2**). Notably, resistance to cefoperazone/sulbactam emerged by day 42, with the minimum inhibitory concentration increasing from 16 to ≥64 µg/mL after 20 days of intravenous treatment. Phylogenetic analysis showed that HD12 clustered with 17 other sequences from human sources in China, derived from various sample types including abdominal fluid (1), blood (5), bronchoalveolar lavage fluid (4), cerebrospinal fluid (1), sputum (3), kidney preservation fluid (2), and one unspecified, while HD12 was the only one obtained from a wound (**Figure 1B**).

Patient 2 was admitted with osteomyelitis with purulent yellow discharge lasting for about one month. Initial cultures were negative, but sinus tract sampling on day 4 yielded *Staphylococcus aureus*, and an incision exudate on day 10 was positive for ABC by culture (isolate HD8). Phylogenetic analysis placed HD8 within a subclade of 10 genomes from six countries across four continents, originating from humans (3), ICU furniture (2), and birds (5). Additionally, none of the twenty sequences in the adjacent subclades were from China, while 55% (11/20) were human-associated (**Supplementary Figure S1A**).

*A. baumannii* isolates HD136 and HD167, obtained from patients 3 and 4 about one year apart, were phylogenetically related (**Supplementary Figure S1B**). Patient 3 was admitted with a fractured left femur after falling from a height. Skeletal traction was performed on the day of admission in the patient ward, and internal fixation was carried out on day 5. Isolate HD136 was cultured from the incision secretion on day 11. Together with other four strains from China, it belonged to a subclade of 17 genomes, all recovered from human across 11 countries of five continents with 71% (12/17) from Asia. Patient 4, admitted for implant removal, had drainage cultivated daily starting on postoperative day 6. HD167, isolated on day 9, belonged to the neighbor subclade of HD136, which predominantly composed of human isolates (10/12), with most originating from China (8/12).

Patient 5 underwent surgery upon admission due to the bleeding left middle finger with open fracture, presenting with a 3-cm irregular ring-shaped laceration and severe contamination. Purulent exudate appeared on day 8, and ABC was identified by culture. *A. baumannii* HD181 was recovered on day 13. In the phylogenetic tree, it clustered within a subclade comprising human-/avian-retracted (8 or 4 out of 14) sequences from diverse geographical regions, including China. The three most closely related isolates were avian-associated, one from Germany and two from Poland (**Supplementary Figure S1C**).

Patient 6 was hospitalized for hip replacement 40 days post injury. ABC was first identified from serosanguinous drainage from the surgical incision 6 days after surgery. Isolate HD239, as the only one from wound exudate, was fitted into a subclade of 13 isolates all collected from China, including eight from humans, one from *Andrias davidianus* (Chinese giant salamander), and two from *Apis mellifera* (honeybee) (**Supplementary Figure S1D**).

Isolates HD11 and HD13 were recovered from patients 7 and 8, respectively, both upon admission. Identified as ABC by culture and *A. pittii* by WGS, these two isolates showed distinct genomic profiles (**Figure 1C**). Patient 7 was hospitalized to remove orthopedic implants in the right ankle one year post surgery, presenting with a 1-cm unhealed wound and viscous yellow drainage. Culture revealed a polymicrobial infection including ABC, *Proteus mirabilis* (extended-spectrum beta-lactamase, ESBL-positive), and *Klebsiella pneumoniae*. HD11 was phylogenetically placed at the edge of *A. pittii* tree with an ANI of 94.6%, but shared a common ancestor with 96% of the 349 A*. pittii* genomes analyzed. It was most closely related to one soil isolate from the USA (GCA_003053325.1) and one human wound isolate from China (GCA_022449185.1) (**Figure 1D**). Patient 8, admitted four days after trauma, was diagnosed with a skin and soft tissue infection. Isolate HD13 clustered within a phylogenetic subclade where approximately 84% (26/31) of strains were human-associated, primarily from the USA (10) and China (9) (**Figure 1E**).

Patient 9 was admitted four hours after trauma with injured left extremity. Upon admission, isolate HD108 was recovered from a heavily contaminated deep wound, along with *Escherichia coli*, and was initially reported as ABC by culture, but later identified as *A*. *soli* by WGS (**Figure 1F**). A total of 35 published *A. soli* genomes fulfilled the phylogenetic analysis criteria of this study. Among the 36 genomes in the *A. soli* phylogenetic analysis, including HD108, 83% (30/36) were from human sources, primarily blood (15) and sputum (11), mostly collected in Japan (12) and China (11). Of note, HD108 was closely related to four human isolates from Japan, two environmental samples from India, and one avian isolate from China.

### 3.2 Genomic and phenotypic analysis of clinical isolates

**Figure 3:**
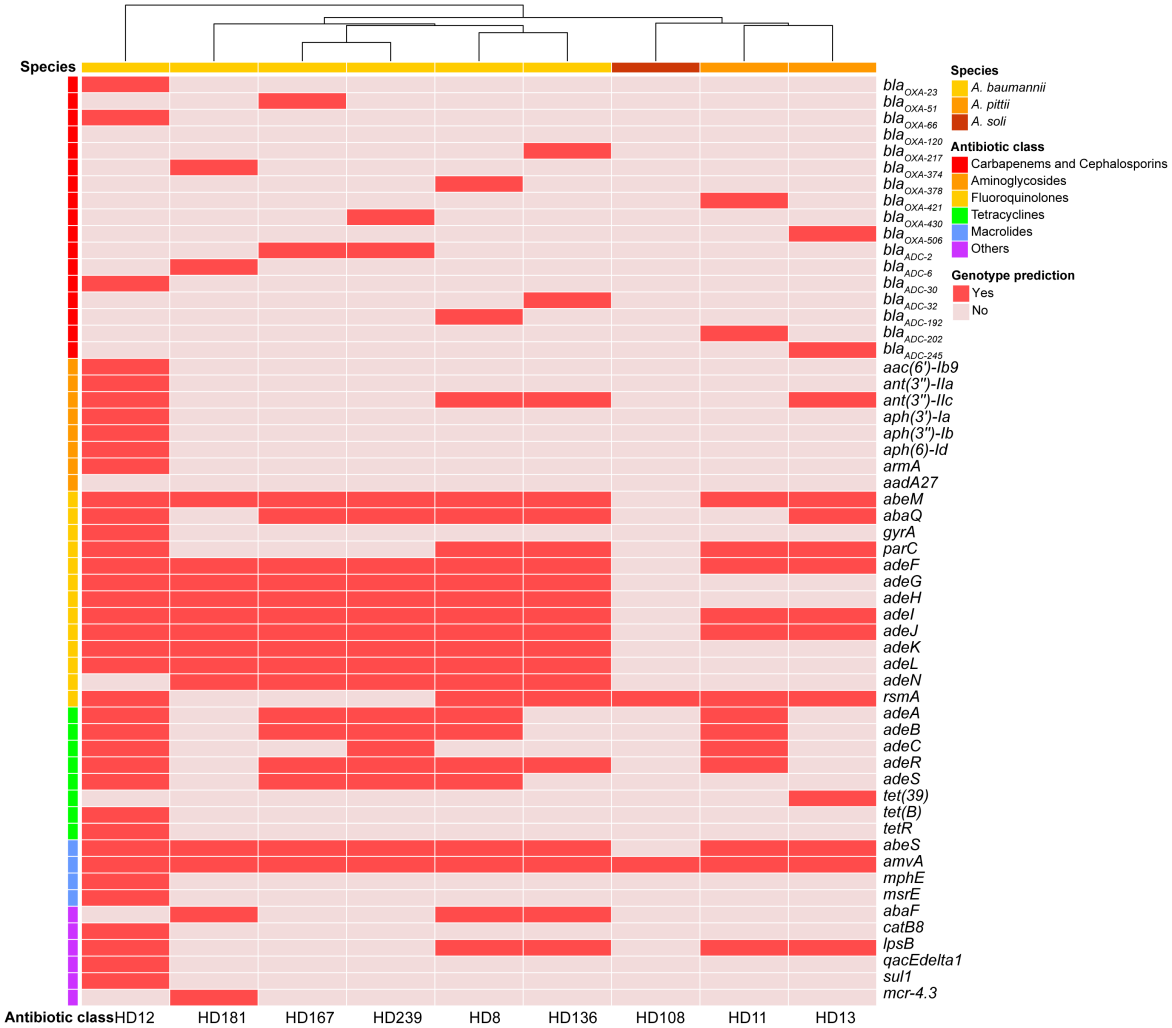
Antibiotic resistance genes identified in the nine isolates. Genes were annotated using CARD (https://card.mcmaster.ca/analyze/rgi) and clustered based on the occurrence frequency of each AMR encoding gene.

Among the six *A. baumannii* clinical isolates (HD8, HD12, HD136, HD167, HD181, and HD239), two *A*. *pittii* isolates (HD11 and HD13), and one *A*. *soli* isolate (HD108), genomic analysis identified no plasmid in any isolate. Intact prophages were detected in all isolates except *A. baumannii* HD8 and *A*. *soli* HD108. Notably, *A. baumannii* HD12, HD136, and *A*. *pittii* HD11 carried ARGs within predicted prophages. In HD11, the *β*-lactamase encoding gene *bla*_OXA-421_ and efflux pump (EP) genes *adeABCR* were located within prophage regions. While other EP genes, *adeL*-*adeFGH* and *adeIJK*, were present in all six *A. baumannii* strains, *adeABC* genes were exclusively detected in HD12 (**Figure 3**).

*A. baumannii* HD136 and HD181 harbored a type I-F CRISPR-Cas system. Furthermore, HD136 contained one compositive transposon carrying quinolone-resistant genes *abaQ* and *adeL*. A clear correlation between genotypic and phenotypic profiles of antimicrobial resistance (AMR) was observed in both *A. baumannii* HD12 and *A. soli* HD108, where HD12 displayed MDR and HD108 appeared susceptible to all antibiotics tested (**Figures 2**, **3**). In contrast, the remaining five *A. baumannii* isolates and the two *A. pittii* strains harbored ARGs conferring resistance to various antimicrobial classes. However, apart from HD239, which was resistant to cefotaxime, they all exhibited intermediate resistance to ceftriaxone only, while *A*. *pittii* HD11 was phenotypically susceptible to all examined drugs (**Figures 2**, **3**).

*β*-lactamase encoding genes, *bla*_OXA_ and *bla*_ADC_, were detected in all isolates except *A*. *soli* HD108. All *A. baumannii* strains carried intrinsic *bla*_OXA-51-like_ genes, while both *A*. *pittii* carried *bla*_OXA-213-like_ alleles (**Figure 3**). Of note, *A. baumannii* HD12 bore both *bla*_OXA-23_, the most common OXA-type carbapenem-hydrolyzing *β*-lactamase, and the intrinsic OXA-51-like *bla*_OXA-66_ (Bush and Bradford, 2020). Insertion sequence IS*Aba1* bracketed *bla*_OXA-23_ and *bla*_OXA-66_ in either opposite or same orientation, and was also present downstream of *bla*_ADC-30_ in the reverse orientation (data not shown). HD181 harbored *bla*_OXA-374_, *bla*_ADC-6_, and *mcr-4.3* with IS*Aba13* located downstream of *bla*_OXA-374_ (data not shown). Virulence factors (VFs) involved in adherence, immune evasion, and stress survival were detected across all species, with minor differences in non-canonical VFs, such as *katAB* and *ureB* (**Supplementary Figure S2**).

Sequence types (STs) of the isolates were shown in **Figure 1**. Worth noting is that ST3401 and ST3402 were newly assigned to *A. baumannii* HD167 and *A*. *pittii* HD11, respectively. Corroborated by its marginalized phylogenetic status (**Figure 1A**), HD181 cannot be assigned a valid ST type because its *gdhB* gene lacked a suitable match in the multi-locus sequence typing scheme of *A. baumannii* on pubMLST.org. Additionally, no ST assignment was available for *A. soli* (**Table 1**).

## 4 Discussion

*A. baumannii* has been attracting great attention globally because of its versatile capability to acquire MDR and to thrive in healthcare environment, particularly against the last-resort treatment option carbapenems (Muller et al., 2023). In contrast, non-*A. baumannii* species are frequently misinterpreted in clinical practice due to the limited discriminatory power of routine diagnostic approaches, which consequently underestimates non-*A. baumannii* infections in the real world (Lauderdale et al., 2014; Almuzara et al., 2015). In this study, all nine *Acinetobacter* isolates were initially identified as members of the ABC by the clinical laboratory, but were subsequently confirmed to belong to three distinct species with unique genomic and phenotypic characteristics.

All six *A. baumannii* cases were likely associated with nosocomial infections, as suggested by the interval between patient admission and sample collection. This inference warrants further validation through targeted environmental sampling. Among them, only HD12, one CRAB strain of the main epidemic ST208, harbored both *bla*_OXA-23_ of higher carbapenemase activity and the intrinsic OXA-51-like *bla*_OXA-66_ of weak carbapenem resistance, in addition to other MDR genes, which indicates limited treatment options. Notably, Patient 1 was positive with CRAB for 26 consecutive days. This prolonged detection, the fact of ICU stay, along with the phylogenetic proximity of HD12 to clinical *A. baumannii* isolates in China carrying similar resistomes, including *bla*_OXA-23_, strongly support possible nosocomial acquisition (**Supplementary Figure S3**) (Liu et al., 2022). The emergence of cefoperazone resistance during treatment underscored the risk of unintentionally accelerating antimicrobial resistance through empiric therapy. As previously reported, upstream IS*Aba1*can upregulate the expression of *β*-lactamase by acting as a promoter for both *bla*_OXA-51-like_ genes and *bla*_ADC_ genes (Bush and Bradford, 2020). The presence and functional relevance of predicted IS*Aba1* flanking both *bla*_OXA_ and *bla*_ADC-30_ in HD12 merit further investigation.

Except for HD12, all the other five *A. baumannii* strains belonged to uncommon ST types. Surprisingly, despite their hospital-associated origin, they exhibited susceptibility to most tested antibiotics. For example, HD8 clustered phylogenetically with strains retrieved from humans, animals, and environment sources, suggesting possible transmission within a One Health framework (Castillo-Ramírez, 2022). HD136 shared a comparable resistance genotype and the common human host with genetically close-related clones across continents, implying ongoing pathogen circulation (**Supplementary Figure S4**). Similar phylogenetic traits were also observed for HD167, HD181, and HD239.

Of particular concern, HD181 harbored chromosomal *mcr-4.3*, a variant of the mobilized colistin resistance gene, which was first described on an *Enterobacter cloacae* plasmid in 2014 exhibiting a silent phenotype due to mutations (Teo et al., 2018). This *mcr-4.3* in HD181 shared considerable genetic similarity with other *A. baumannii* sequences from diverse sources in different geographic regions (**Supplementary Figure S5**). The chromosomal location allows vertical transmission, while horizontal transfer could occur via plasmid mobilization. Given the wide spread of *mcr-4.3* among bacterial genera, its transmission between plasmid and chromosome, and the possibly restored colistin resistance under antibiotic pressure, strains alike HD181 pose a serious threat as a silent reservoir and transmitter for potential colistin resistance within clinical and community settings (Zhang et al., 2019).

All three non-*A. baumannii* isolates appeared to be community-acquired. Both *A. pittii* strains carried ARGs conferring resistance to multiple antibiotic classes, yet remained largely susceptible phenotypically. *A. soli* HD108 also displayed drug susceptibility. Genomic analysis indicated that 67% of publicly available *A. soli* genomes, including HD108, had resistance genes *amvA* and *rsmA* only, although seven clones from China were found to carry metallo-*β*-lactamases VIM-11, IMP-1, NDM-1, and other ARGs (data not shown). These findings indicated that MDR features, common in *A. baumannii*, have yet to become prevalent in *A. pittii* and *A. soli*. The phylogenetic distinctiveness of *A. pittii* HD11 is compelling for further investigation.

Timely and precise identification of causative pathogens, especially those with AMR or MDR, is critical for clinical treatment decision-making. Medical chart review revealed that clinicians lean heavily on empirical and broad-spectrum antibiotics before the laboratory reports pathogen identification and susceptibility profiles. Among the nine cases interrogated in the current study, only the strain HD12 from Patient 1 demonstrated MDR, requiring alternative therapeutic strategies. However, in reality, all patients except Patient 4 received cephalosporin treatment upon reporting ABC-positive results. First-generation cephalosporin cefazolin was administrated to seven patients, except for Patient 4 (*A. baumannii* HD167) and Patient 9 (*A. soli* HD108). The fluoroquinolone levofloxacin was prescribed for six patients, excluding Patients 7 and 8 with *A. pittii*, and Patient 9 with *A. soli* (**Table 2**). For the rare *A. pittii* and *A. soli* infections, cefazolin was used in Patients 7 and 8, while Patient 9 was treated with ceftazidime after specimens were reported ABC positive. In fact, all three isolates were susceptible to nearly all tested drugs. These findings highlighted potential limitations in clinical practice at this regional hospital. However, the inability to accurately identify *Acinetobacter* species other than ABC may have contributed to inappropriate antibiotic administration in the reported cases, driven by generalized protocols designed for clinically significant drug-resistant *A. baumannii*, potentially leading to unnecessary antimicrobial exposure. Such excessive or inappropriate antibiotic use may accelerate the development and dissemination of AMR within the healthcare system and potentially into the surrounding community. Therefore, accurate species-level identification of *Acinetobacter* is of critical clinical importance for guiding appropriate therapy and minimizing the risk of antibiotics misuse.

**Table 2:** Antibiotic treatments of the enrolled patients in the current study.

Patient	Isolate	Antibiotic treatment				Patient	Isolate	Antibiotic treatment			
		Drugs	Start	End	Duration (days)			Drugs	Start	End	Duration (days)
**P1**	HD12 *A. baumannii*	Piperacillin/Tazobactam	2021-02-18	2021-02-25	8	**P5**	HD181 *A. baumannii*	Cefazolin	2023-08-04	2023-08-14	11
	CRAB	Cefazolin	2021-02-25	2021-03-10	14			Levofloxacin	2023-08-14	2023-08-21	8
		Tinidazole	2021-03-09	2021-03-16	8	**P6**	HD239 *A. baumannii*	Cefazolin	2024-06-21	2024-06-22	1
		Levofloxacin	2021-03-09	2021-03-16	8			Levofloxacin	2024-06-29	2024-07-04	6
		Cefoperazone/Sulbactam	2021-03-10	2021-03-16	7			Ceftazidime	2024-07-03	2024-07-04	2
		Ciprofloxacin	2021-03-16	2021-04-13	29			Cefoperazone/Sulbactam	2024-07-04	2024-07-12	9
		Cefoperazone/Sulbactam	2021-03-16	2021-04-13	29			Gentamicin	2024-07-05	2024-07-18	14
		Cefazolin	2021-04-20	2021-04-26	7			Cefoperazone/Sulbactam	2024-07-13	2024-07-15	3
**P2**	HD8 *A. baumannii*	Cefazolin	2021-01-31	2021-02-05	6			Levofloxacin	2024-07-15	2024-07-27	13
		Clindamycin	2021-02-05	2021-02-12	8	**P7**	HD11 *A. pittii*	Cefazolin	2021-02-24	2021-02-25	2
		Levofloxacin	2021-02-12	2021-02-13	2			Ceftazidime	2021-02-28	2021-03-05	6
**P3**	HD136 *A. baumannii*	Cefazolin	2022-10-05	2022-10-14	10	**P8**	HD13 *A. pittii*	Cefazolin	2021-09-07	2021-09-12	6
		Levofloxacin	2022-10-17	2022-10-30	14	**P9**	HD108 *A. soli*	Ceftazidime	2022-07-28	2022-08-01	5
**P4**	HD167 *A. baumannii*	Levofloxacin	2023-10-29	2023-11-09	12						

Furthermore, discrepancies between genotypic and phenotypic AMR profiles reinforce the necessity of routine susceptibility testing and raise concerns regarding silent ARGs. These genes, located on chromosome or mobile genetic elements, may act as possible reservoirs for unpredictable AMR dissemination among *Acinetobacter* spp. or even other genera, which poses serious challenges for clinical management. As expected, the intrinsic *β*-lactamase genes *bla*_ADC_, *bla*_OXA-51-like_, and *bla*_OXA-213-like_ were detected in most isolates, except in *A. soli*, while only the ICU-acquired *A. baumannii* HD12 demonstrated strong phenotypic antibiotic resistance.

The current study was exploratory with several limitations, including a small sample size, single-center design, limited clinical information, and lack of environmental sampling. Only descriptive results were presented without statistical analysis. Nevertheless, it’s still appealing that these orthopedic infections involving diverse *Acinetobacter* species, including phylogenetically distant strains of the same species, were identified from a small number of patients within a single hospital department covering a geographically confined area. Future investigations with larger cohorts and environmental surveillance are warranted. But considering the devastating consequences of MDR in orthopedic infections and the diagnostic challenges posed by *Acinetobacter* speciation, the observed *Acinetobacter* diversity may reflect a broader and underappreciated epidemiological landscape of non-*A. baumannii* species (Hischebeth et al., 2015; Karakonstantis and Kritsotakis, 2021). To avoid redundancy, only one ABC isolate obtained from each patient was selected for WGS to represent the diversity, which prevented us from exploring the mixed infection or the emergence of AMR mutations. Additionally, concurrent environmental sampling should be considered in future studies to trace potential sources of nosocomial dissemination.

Based on both phenotypic and genotypic analyses, this study investigated nine orthopedic infection cases caused by *Acinetobacter* species, including six *A. baumannii*, two *A. pittii*, and one *A. soli*. The results revealed overtreatment of several cases, particularly those infected with non-*A. baumannii* species. Silent AMR genes were present across *Acinetobacter* species. The discovery of novel sequence types highlighted the existence of unrecognized genetic diversity. Altogether, these exploratory findings tend to underscore the importance of accurate species-level differentiation of non-*A. baumannii* strains and emphasize the need of comprehensive epidemiological surveillance of *Acinetobacter* species, particularly in low-resource settings. In conclusion, improved diagnostic precision and properly informed antimicrobial stewardship may be crucial to mitigate the emergence and spread of AMR in both nosocomial settings and general communities.

## Data Availability

The datasets presented in this study can be found in online repositories. The names of the repository/repositories and accession number(s) can be found in the article/**Supplementary Material**. Genome assemblies in this study have been deposited in NCBI database under BioProject PRJNA1080311.
