# Supplementary material for: Orthopedic infections associated with distinct Acinetobacter strains in rural area of Qingdao, China
Source: Front Cell Infect Microbiol. 2025 Jul 30;15:1601779. doi: 10.3389/fcimb.2025.1601779 (PMC12343611; doi:10.3389/fcimb.2025.1601779)
Supplement: Supplementary file 1 [file DataSheet1.docx]

Supplementary Material

# Supplementary Figures


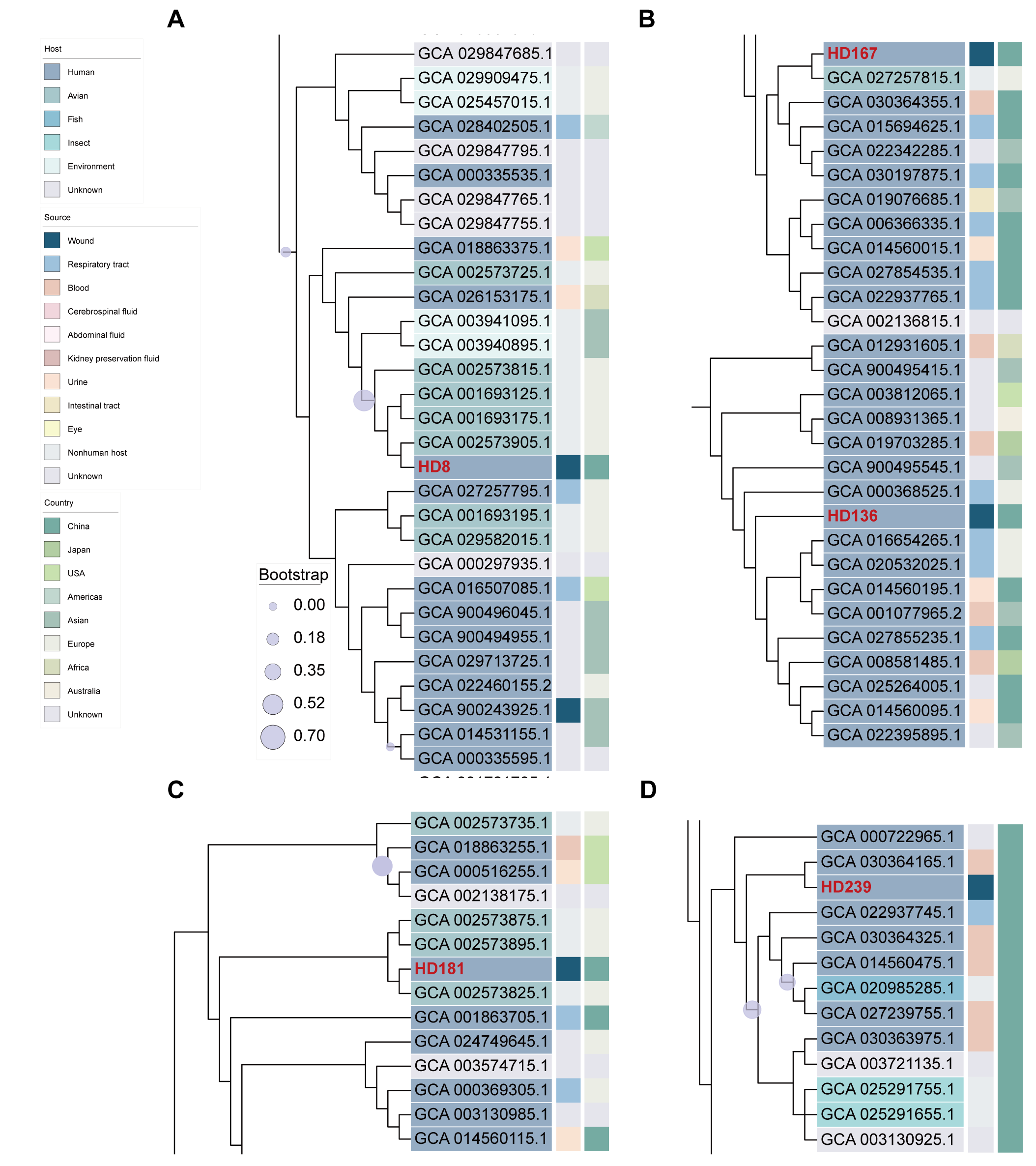


**Supplementary Figure 1.** Corresponding phylogenetic subclades of *A. baumannii* isolates HD8 (A), HD136 (B), HD167(B), HD181 (C), and HD239 (D).


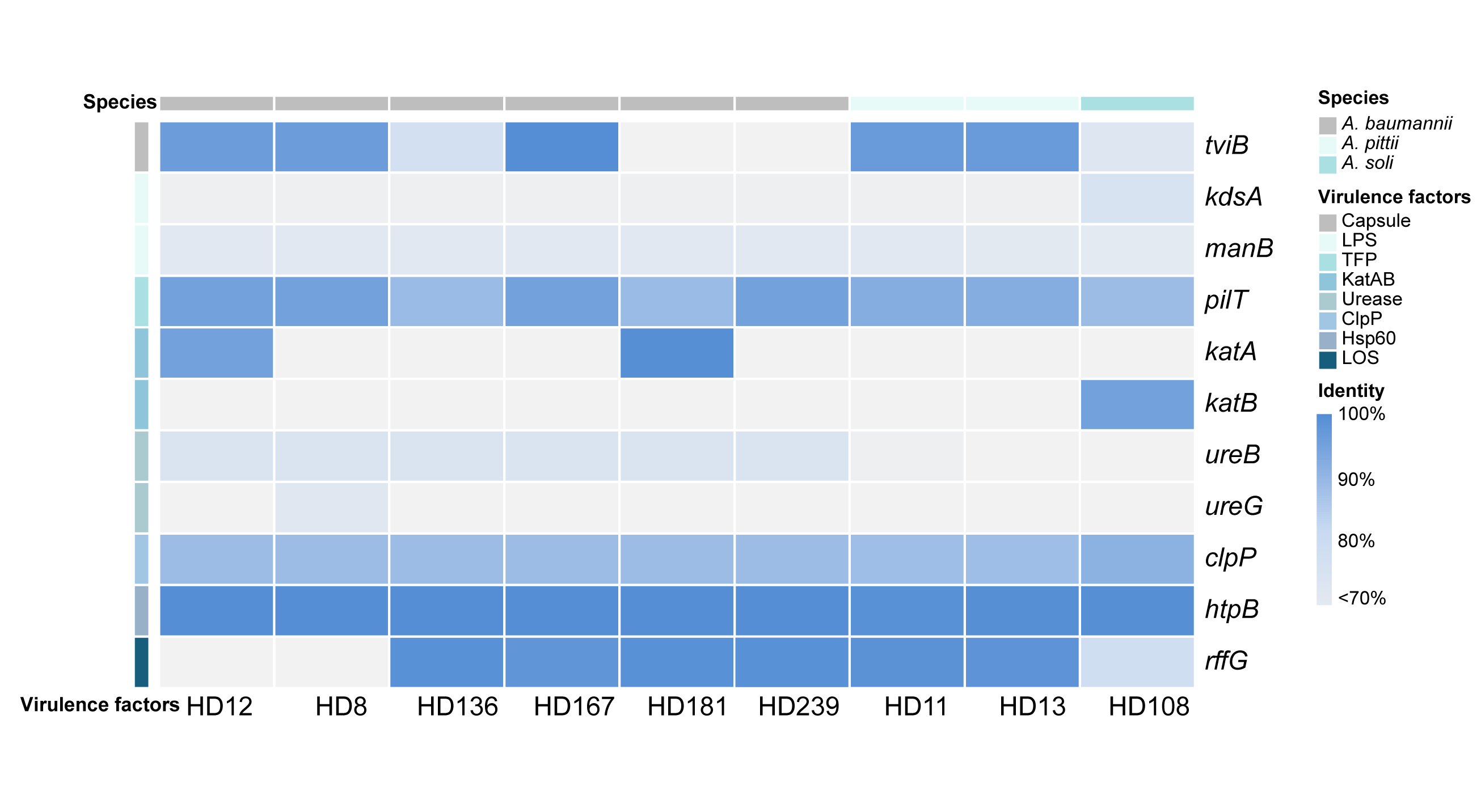


**Supplementary Figure 2.** Virulence genes of all nine isolates predicated by VFDB with sequence coverage and identity both set as ≥70% (https://www.mgc.ac.cn/VFs/main.htm). LPS, Lipopolysaccharide; TFP, Type IV Pili; Los, Lipo-oligosaccharide.


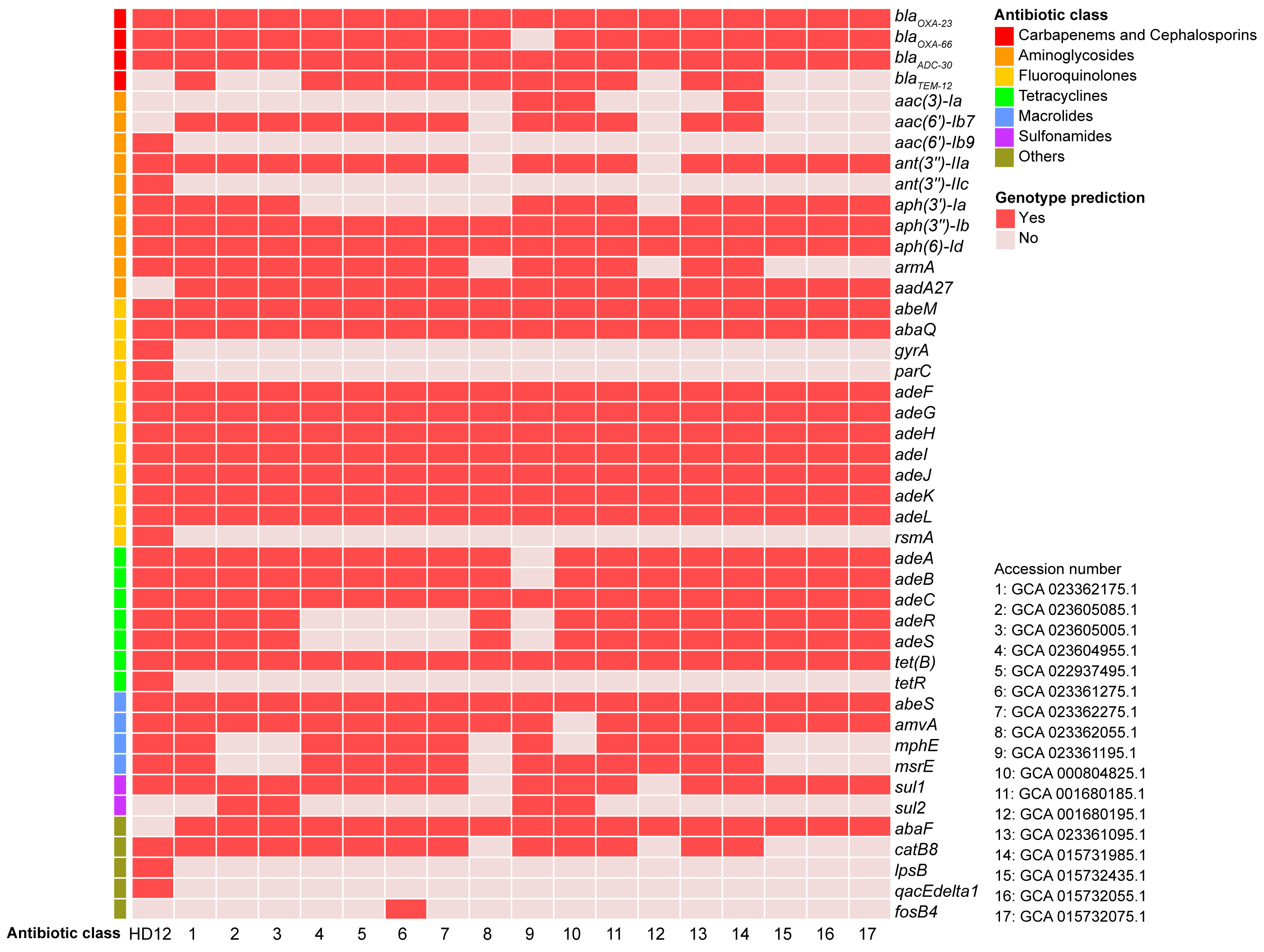


**Supplementary Figure 3.** Antibiotic resistance genes predicted through CARD for *A. baumannii* HD12 and its phylogenetic subgroup members.


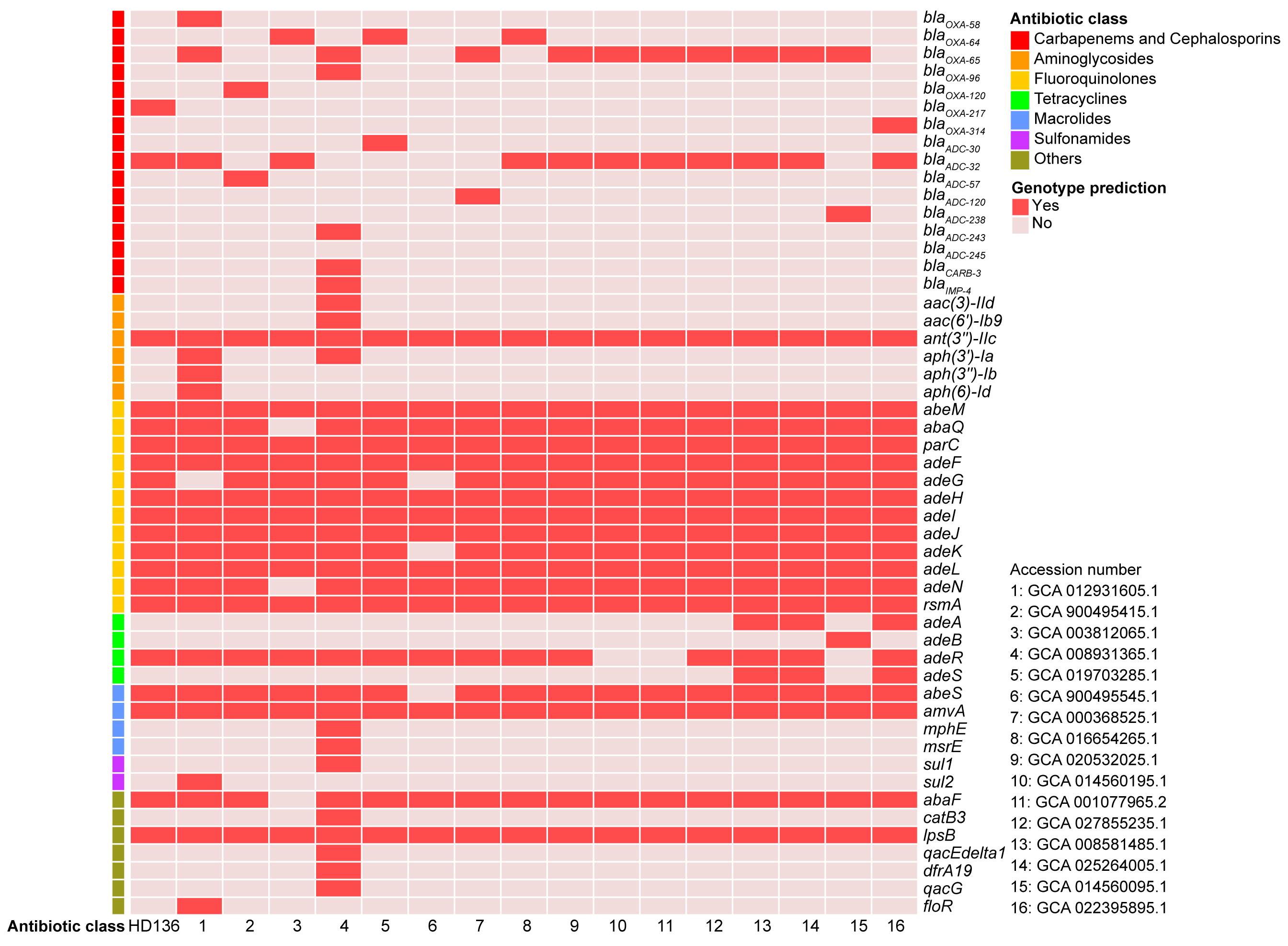


**Supplementary Figure 4.** Antibiotic resistance genes of *A. baumannii* HD136 and other strains in the same phylogenetic subclade identified via CARD.





**Supplementary Figure 5.** Neighboring genetic context of *mcr-4.3* in HD181 and structural comparation with representative *A. baumannii* sequences reported previously. The arrows indicate the position and the transcription directions of genes, which are colored based on their predicted functions. Nucleotide sequence identity ≥70% is depicted in the change of color gray.
